# Relationship between Non-Hodgkin’s lymphoma and blood levels of Epstein-Barr Virus in children in north-western Tanzania: a case control study

**DOI:** 10.1186/1471-2431-13-4

**Published:** 2013-01-07

**Authors:** Rogatus Kabyemera, Nestory Masalu, Peter Rambau, Erasmus Kamugisha, Benson Kidenya, Anita De Rossi, Maria Raffaella Petrara, Damas Mwizamuholya

**Affiliations:** 1 Department of Pediatrics and Child Health, Bugando Medical Centre, P.O. Box 1370, , Mwanza, Tanzania; 2 Department of Oncology, Bugando Medical Centre, P.O. Box 1370, , Mwanza, Tanzania; 3 Department of Pathology, Catholic University of Health and Allied Sciences – Bugando, P.O. Box 1464, , Mwanza, Tanzania; 4 Department of Biochemistry and Molecular Biology, Catholic University of Health and Allied Sciences – Bugando, P.O. Box 1464, , Mwanza, Tanzania; 5 Department of Surgery, Oncology and Gastroenterology, Section of Oncology and Immunology, University of Padova, Unit of Viral Oncology, IOV-IRCCS, Via Gattamelata 64, 35128, Padova, Italy

**Keywords:** Non-Hodgkin’s Lymphoma, Children, HIV, EBV

## Abstract

**Background:**

Non-Hodgkin’s Lymphomas (NHL) are common in African children, with endemic Burkitt’s lymphoma (BL) being the most common subtype. While the role of Epstein-Barr Virus (EBV) in endemic BL is known, no data are available about clinical presentations of NHL subtypes and their relationship to Human Immunodeficiency Virus (HIV) infection and Epstein Barr Virus (EBV) load in peripheral blood of children in north-western, Tanzania.

**Methods:**

A matched case control study of NHL subtypes was performed in children under 15 years of age and their respective controls admitted to Bugando Medical Centre, Sengerema and Shirati district designated hospitals in north-western, Tanzania, between September 2010 and April 2011. Peripheral blood samples were collected on Whatman 903 filter papers and EBV DNA levels were estimated by multiplex real-time PCR. Clinical and laboratory data were collected using a structured data collection tool and analysed using chi-square, Fisher and Wilcoxon rank sum tests where appropriate. The association between NHL and detection of EBV in peripheral blood was assessed using conditional logistic regression model and presented as odds ratios (OR) and 95% confidence intervals (CI).

**Results:**

A total of 35 NHL cases and 70 controls matched for age and sex were enrolled. Of NHLs, 32 had BL with equal distribution between jaw and abdominal tumour, 2 had large B cell lymphoma (DLBCL) and 1 had NHL-not otherwise specified (NHL-NOS). Central nervous system (CNS) presentation occurred only in 1 BL patient; 19 NHLs had stage I and II of disease. Only 1 NHL was found to be HIV-seropositive. Twenty-one of 35 (60%) NHL and 21 of 70 (30%) controls had detectable EBV in peripheral blood (OR = 4.77, 95% CI 1.71 – 13.33, p = 0.003). In addition, levels of EBV in blood were significantly higher in NHL cases than in controls (p = 0.024).

**Conclusions:**

BL is the most common childhood NHL subtype in north-western Tanzania. NHLs are not associated with HIV infection, but are strongly associated with EBV load in peripheral blood. The findings suggest that high levels of EBV in blood might have diagnostic and prognostic relevance in African children.

## Background

Non-Hodgkin’s Lymphomas (NHLs) are common in African population and several histological subtypes with different clinical presentations and treatment options have been described based on the WHO classification of the mature B-cell neoplasm [1]. Worldwide, reports on the incidence of lymphomas are variable, with 60% of all childhood lymphomas being classified as NHL, representing 8% of all childhood malignancies [2-4].

In Equatorial African countries, the most common subtype of NHL in children is endemic Burkitt’s lymphoma (BL) [5-7], possibly due to the high prevalence of malaria, Human Immunodeficiency Virus (HIV) and Epstein Barr Virus (EBV) infections in this region [5]. The magnitude of NHL in Tanzania is unknown because of the lack of a national cancer registry. However, a recent clinical study in north western Tanzania reported an incidence of endemic BL of 4.2 per 100,000 [8].

The clinical presentation of childhood NHL depends primarily on the subtype and the site of involvement. Approximately 70% of children who present with NHL have advanced disease (i.e., stage III or IV) and/or have metastatic involvement including bone marrow and central nervous system (CNS) [9-11].

Endemic BL is commonly seen in children aged 4–9 years [12] and presents with a painful jaw tumour, loose or disarranged teeth [13] and less commonly with abdominal mass or paraspinal disease. However, few African studies reported a predominance of abdomen over jaw manifestation [14,15]. Bone and bone marrow involvement is unusual. Sporadic childhood BL is more evident in white, male children and commonly presents with enlarged lymph nodes and abdominal tumours [16].

Childhood lymphoblastic lymphoma is commonly manifested by either intrathoracic and/or mediastinal mass, with various forms of respiratory distress, bilateral pleural effusions and hepatosplenomegaly [17] and rarely involve both the CNS and bone marrow [18]. Large B-cell lymphoma presents with an abdominal or mediastinal mass, enlarged lymph nodes and rarely involve both the CNS and bone marrow [2].

In African children, the association between NHL and HIV is uncertain. Two studies from Uganda and Rwanda described a relationship between NHL and HIV infection [19,20] but most studies, including those with cases confirmed by histology, reported no association between HIV and BL [21-23].

In paediatric patients, studies have reported an association between EBV and NHL in 21-25% of cases [24,25] and even 100% in children with primary and acquired immunodeficiency [26]. Moreover, 95% of endemic BL have been reported to be EBV positive compared to 15 to 20% of the sporadic BL cases [4,7,8]. Both EBV type 1 and EBV type 2 have been associated with BL, with a predominance of EBV type 1 [17,27-30]. However, very little is known about EBV load in peripheral blood of African children with NHL. A study in samples from Malawi reported higher EBV DNA loads in peripheral blood of children with BL compared to their controls [31].

The aim of this study was to describe the subtypes and clinical presentations of NHL in children in north-western Tanzania and their relationship with HIV infection and EBV load in peripheral blood.

## Methods

### Study area and population

This was a case control study, done in paediatric and oncology wards of the three clinical centres in north-western Tanzania, namely Bugando Medical Centre (BMC), Sengerema and Shirati designated district hospitals in Mwanza and Mara regions, between September 2010 and April 2011. BMC is the zonal consultant and teaching hospital located in Mwanza city; it has a 900 bed capacity and it serves around 14 million people from 6 regions of the Lake Zone, namely Mwanza, Kagera, Shinyanga, Tabora, Mara and Kigoma. About five children with tumours are admitted monthly for investigation and treatment. Sengerema and Shirati DDH have approximately 300 and 200 bed capacities, respectively, and they each serve around 30 and 15 cases of childhood lymphomas every year.

Cases were enrolled if they were ≤15 years of age with a diagnosis of NHL and if consent from caretakers to participate in the study was obtained. Controls were included if they were admitted to the general paediatric wards with non-malignant conditions and were matched for age and sex with NHL cases. Children with malignancies that overlapped NHL, e.g. acute lymphoblastic leukaemia, and children with relapse of NHL were excluded from the study.

### Sample size estimation

Sample size was calculated using OpenEpi software, version 2 [32], in which Fleiss formulae for case control studies was applied to determine the appropriate sample size for a power of 0.8 and significance level of 0.05. The minimum sample size for cases was 1/3 (33) and for controls was 2/3 (66) making the minimum sample size of 99.

### Study clinical procedures

All consecutive NHL cases were recruited until the required sample size was attained, whereas simple randomization was done to select two controls per each case from all eligible patients. The investigator or trained research assistant either admitted the patient or reviewed the files of all eligible children within 2 days of admission and using a structured data collection tool, demographic data, clinical data and required investigation results were recorded.

To collect samples for cytological or histological diagnosis of NHL subtypes, fine needle aspiration of the tumour was carried out, using a needle of 25 or 27 gauge and a 20 ml disposable plastic syringe, under sedation with midazolam (0.1 mg/kg) and ketamine (1 mg/kg), or tissue biopsy of the tumour was carried out under general anaesthesia. In the case of an abdominal mass, an ultrasound scan was used to guide the needle into the tumour. Fine needle aspirate was smeared between two standard microscope slides with one slide immediately fixed with 95% alcohol and the other air dried and then transported in a slide carrier to the histopathology laboratory. Alcohol fixed smears were stained by Papanicolaus stain and air dried slides were prepared and stained with Giemsa.

Tissue biopsies were taken in the operating theatre, fixed immediately with 10% formalin and then transported to the pathology laboratory. Histology slides were stained with haematoxylin and eosin. At least two pathologists examined the slides under light microscopy and children with confirmed NHL diagnosis were enrolled in this study.

Lumbar puncture was performed in order to obtain cerebral spinal fluid (CSF) for cytological evaluation. A sterile 22, 38 mm gauge lumbar puncture needle (BD spinal needle) was inserted between the fourth and fifth lumbar vertebrae and the stylet withdrawn to allow about 5 ml of CSF to freely flow through the needle into the sterile tube which was then sent to the pathology laboratory to detect the presence or absence of cancer cells. Presence of cancer cells in the CSF indicated involvement of the CNS.

Bone marrow aspiration was performed, using a 13,5 cm gauge bone marrow needle (Jamshidi bone marrow aspiration needle), under sedation with midazolam and ketamine; 1, 2 ml of bone marrow were aspirated into the syringe and then smeared on 8 slides (4 fixed with alcohol and 4 without fixation) that were air dried and sent to the pathology laboratory for cytological analysis. Presence of lymphoma cells indicated bone marrow involvement.

NHL staging was done using the St. Jude’s staging of childhood NHL [33].

Blood was drawn from the median cubital vein and collected in plain and EDTA bottles and then sent to the haematology and biochemistry laboratories for analysis of complete blood count and erythrocyte sedimentation rate, serum creatinine, alanine amino transferase and aspartate amino transferase.

The Tanzania national algorithm for HIV testing was followed to determine the HIV status of both cases and controls, using two antibody tests, the SD Bioline (SD Standard Diagnostics, Inc) and the Determine (Determine HIV 1/2 Serum/Plasma Assays); the second test was carried out in samples reactive to the first test. Those samples that were reactive to the first and second tests were considered to be HIV positive. Discordant results were subjected to a third test, Unigold (The Trinity Biotech PLC) [34]. Counselling on HIV testing was done and consent was sought prior to testing. Among HIV positive children, CD4 cell count was measured using Facscount (BD, San Jose, California, USA).

### Blood collection and DNA elution

Approximately 50 μl of blood was spotted onto each circle of Whatman 903 filter paper and then dried at room temperature overnight. Dried Blood Spots (DBS) were stored in individual ziplock bags containing a desiccant, and sent to the laboratory of the Department of Surgery, Oncology and Gastroenterology, Section of Oncology and Immunology, University of Padova, Unit of Viral Oncology-IOV IRCCS, Italy. From each 50 μl DBS, three 3 mm-diameter circles, equivalent to 5 μl of whole blood each for a total of 15 μl whole blood, were used to extract DNA with the DNA Micro Kit (Qiagen, Hilden, Germany) and resuspended in 50 μl final volume.

### EBV-DNA quantification

To control the ability of the eluted DNA from DBS to be amplified, 5 μl of DNA from each sample were amplified for the human telomerase reverse transcriptase (hTERT) located in the 5p15.33 (Gene Bank accession: AF128893), employed as housekeeping gene. Amplification was carried out as previously described [35]. A quantitative method based on Multiplex Real-Time PCR assay was performed to quantify EBV type 1 and EBV type 2, using a primer pair for the EBNA2 gene [36,37] and the probes designed to discriminate between EBV type 1 and EBV type 2 ( EBV type 1: 5'-FAM-AAT CCT CCT ACC CTC TCT TTA TGC CAT GTG TGT-TAMRA-3'; EBV type 2: 5'-Cy5 TGG GCT GTT AGT AGG GT-BBQ-3') [38]. A standard reference curve was obtained by five-fold serial dilution of two amplicons, one for EBV type 1 and the other for EBV type 2, and amplification was carried out, as already detailed [38]. The multiplex assay showed a dynamic range from 5 to 2×105 copies. The results were expressed as EBV-DNA copies/ml. In those who had both EBV 1 and 2, viral levels were added and their total viral loads were analysed to investigate the association between NHL and EBV viral load.

### Statistical analysis

Data were screened and edit checks were done to minimize data entry errors. Data were analysed with the STATA 11 software (College Station, Texas, USA). Categorical variables were summarized as percentages and were analysed by Chi-square or Fisher’s exact tests where appropriate. Continuous variables were summarized as mean (standard deviation) or median (range) where appropriate. EBV-DNA levels of cases and controls were compared using Wilcoxon rank sum (Mann–Whitney) test. The association between NHL and EBV was investigated using conditional logistic regression model. Odds ratios (OR) and 95% confidence intervals (CI) were determined by maximum likelihood estimation. The data were considered significant if the p-value was < 0.05.

### Ethical considerations

Ethical approval was obtained from the Joint Bugando Medical Centre/Bugando University College of Health Sciences research and publication committee and guidelines on ethical requirement for conducting research and sample transportation outside the country were adhered to. Confidentiality was assured and the study did not interfere with the decision of the attending physician in case of absence of the investigator.

## Results

### Patient characteristics of NHL cases and controls

35 NHL cases and 70 controls matched for age and sex were enrolled in this study. Among the NHL patients, 21 (60%) were females and 14 (40%) were males (*x*^2^ = 0.92, *p*-value = 0.337). Age at presentation ranged from 3 to 14 years with a peak age of 5 to 9 years (68.57%) and a mean age of 7.48 years (SD +/− 2.77).

Of the 35 NHL cases, 32 patients had a diagnosis of BL, 2 had diffuse large B cell lymphoma (DLBCL) and 1 had NHL-not otherwise specified (NHL-NOS). The controls were diagnosed with sickle cell anaemia (10, 14.3%), severe pneumonia (8, 11.4%), gastroenteritis (5, 7.1%), congenital heart diseases (5, 7.1%), urinary tract infections (5, 7.1%), rheumatic heart diseases (5, 7.1%), malnutrition (5, 7.1%), tuberculosis (4, 5.7%), nephrotic syndrome (3, 4.3%) and others infections or bleeding conditions (20, 28.8%).

### Clinical presentations of NHL cases

The primary tumour site was recorded in all cases. Of the 32 BL cases, jaw and abdomen had an equal distribution; 16 cases (50%) in each tumour site. One BL patient had both abdominal and jaw involvement. Lymph nodes were enlarged in all non-BL NHL cases as the primary tumour site and these involved submandibular, axillary, cervical and inguinal lymph nodes. Axillary and inguinal lymph nodes were enlarged in two BL cases. Fever was reported in 11 cases (31.4%) and anaemia in 28 cases (80%), of which 3 (10.7%) had severe anaemia (Hb ≤ 5 g/dl).

Bone marrow was involved in the patient with NHL-NOS, whereas CNS presentation occurred in one BL patient who was a 7 years old boy and presented with a jaw tumor involving the left eye and weakness of the lower limbs. He was HIV negative but EBV positive and had cancer cells in the CSF. The majority of BL cases (19 of 32) had an early presentation (stage I or II), while the DLBCL and NHL-NOS had a late presentation (stage III and IV) (Table 1). Analysis of fever, anaemia and lymph node enlargement showed a significant association between fever and late stage presentation (p = 0.035).

**Table 1:** Clinical and demographic features of NHL patients

	**NHL subtypes**			**Total**
	**BL**	**DLBCL**	**NHL**-**NOS**	
Number of cases	32/35 (91.4%)	2/35 (5.7%)	1/35 (2.9%)	35/35 (100%)
Age				
1-4	5/32 (15.6%)	0/2 (0%)	0/1 (0%)	5/35 (14.3%)
5-9	21/32 (65.6%)	2/2 (100%)	1/1 (100%)	24/35 (68.6%)
10-15	6/32 (18.8%)	0/2 (0%)	0/1 (0%)	6/35 (17.1%)
Sex				
Male	14/32 (43.8%)	0/2 (0%)	0/1 (0%)	14/35 (40%)
Female	18/32 (56.2%)	2/2 (100%)	1/1 (100%)	21/35 (60%)
Primary tumour site				
Jaw	16/32 (50%)	0/2 (0%)	0/1 (0%)	16/35 (45.7%)
Abdomen	16/32 (50%)	0/2 (0%)	0/1 (0%)	16/35 (45.7%)
Lymph Node	0/32 (0%)	2/2 (100%)	1/1 (100%)	3/35 (8.6%)
CNS involvement	1/32 (3.1%)	0/2 (0%)	0/1 (0%)	1/35 (2.9%)
^a^Bone marrow involvement	0/11 (0%)	0/11 (0%)	1/11 (9.1%)	1/11 (9.1%)
Fever	9/32 (28.1%)	1/2 (50%)	1/1 (100%)	11/35 (31.4%)
Anaemia	26/32 (81.3%)	1/2 (50%)	1/1 (100)	28/35 (80%)
Clinical stage				
Early (I/II)	19/32 (59.3%)	0/2 (0%)	0/1 (0%)	19/35 (54.3%)
Late (III/IV)	13/32 (40.7%)	2/2 (100%)	1/1 (100%)	16/35 (45.6%)

^a^ Bone marrow sampling was done in 11 only cases because of unavailability of facilities in two study sites.

### Association of NHL with HIV infection

Only 1 NHL case and two controls were found to be HIV-seropositive. No significant association was found between NHL and HIV infection.

### Association of NHL with EBV infection, EBV subtypes and viral load

Of the 105 DBS samples, 42 (40.0%) were found to be positive for EBV-DNA by PCR; therefore 42 subjects had detectable levels of EBV in their peripheral blood. Of this subgroup of EBV positive subjects, 21 cases and 21 controls (Table 2), 27 (64.3%) had EBV type 1, while 8 (19.0%) had both subtypes (EBV type 1 and 2). There was no significant difference between EBV subtypes and either age (Fisher’s exact test = 1) or sex (fisher exact test = 0.213). twenty one of 35 (60%) NHL patients had EBV detectable in peripheral blood compared to 21 of 70 (30%) controls; a significant association was found between NHL and EBV (OR = 4.77, 95% CI = 1.71 – 13.33, p -value = 0.003) (Table 2).

**Table 2:** Frequency of EBV detection and EBV load in NHL patients and controls

	**NHL**		**p**-**value**
	**Cases**	**Controls**	
EBV-positive detection in blood	21/35 (60%)	21/70 (30%)	0.003
EBV sub-type			
EBV-1	11/21 (52%)	16/21 (76%)	
EBV-2	5/21 (24%)	2/21 (10%)	
EBV type 1 & 2	5/21 (24%)	3/21 (14%)	
EBV viral load count Median (range) copies/ml	4720 (988– 6250164)	2525 (1290–19452)	0.024

EBV levels were not normally distributed. Hence, a two sample Wilcoxon rank sum test (Mann–Whitney test) was used to analyse the median viral load distribution between cases and controls. This analysis showed a significantly higher median viral load in cases compared to controls (p-value = 0.024) (Table 2).

Analysis of cases revealed no significant association between EBV status and demographic characteristics or clinical presentation (Table 3).

**Table 3:** EBV status and demographic characteristics and clinical presentations in NHL cases

	**EBV**-**DNA positive**	**EBV**-**DNA negative**	**p**-**value**
Age			
1-4 years	2 (40%)	3 (60%)	0.813
5-9 years	17 (70.8%)	7 (29.2%)	
10-14 years	2 (33.3%)	4 (66.7%)	
Sex			
Male	10 (71.4%)	4 (28.6%)	0.22
Female	11 (52.4%)	10 (47.6%)	
NHL subtype			
BL	19 (59.4%)	13 (40.6%)	0.652
Non-BL	2 (66.7%)	1 (33.3%)	
Primary anatomical site			
Jaw	7 (43.8%)	9 (56.2%)	0.247
Abdomen	12 (75%)	4 (25%)	
Lymph nodes	2 (66.7%)	1 (33.3%)	
Clinical stage			
Early	10 (52.6%)	9 (47.4%)	0.332
Late	11 (68.6%)	5 (31.4%)	

## Discussion

In this study, the most common subtype of NHL was found to be BL, a finding similar to previously reported studies [7]. This frequency might be due to the causal relationship between BL and co-infections with malaria and EBV in the north-western regions of Tanzania, which are considered part of the “lymphoma belt” of Africa. BL should therefore be highly suspected in cases of childhood NHL.

The common age at presentation in this study was similar to previous studies, with the majority of patients ranging from 5 to 9 years [12,13]. However, conversely to other studies [2,3] females were more affected than males; this might have been due to the small number of cases reported in this study. Nevertheless, there was no statistically significant relationship between NHL and gender.

The majority of patients in this study were in an early stage of disease at presentation, possibly due to ongoing sensitization programmes and an increase in the number of hospitals that provide free cancer treatment in the Lake zone regions in recent years, compared to the past treatment modalities for NHL cases [2,9-11]. The increased awareness of cancer management, easier accessibility and better healthcare provided by these hospitals today may have sensitized more patients to present early for treatment.

Jaw and abdomen were the most common primary tumour sites in patients with BL. While other studies reported more jaw BL in African children [14], we found no predominance of jaw over abdominal presentation. One reason for more jaw BL cases might be an easier access to jaw tumours for diagnostic purposes compared to abdominal tumours which require more expertise and diagnostic facilities than were not available in the past.

Lymph nodes were involved in all non-BL NHL subtypes. Only 1 patient presented with CNS disease, which is more commonly observed in sporadic BL [11,14]. As previously reported [14], bone marrow involvement was rare. However, a Brazilian study reported 23% of cases with primary bone marrow disease [11]. We could not rule out a possible underestimation since bone marrow sampling was not done in all cases because of unavailability of facilities in two sites.

HIV is believed to act as a trigger in the causation or progression of some cancers because of its ability to cause immune depression. Only one case and two controls were found to be HIV-seropositive and, in agreement with previous observations [21-23], this study did not find any significant association between childhood NHL and HIV infection.

A statistically significant association was found between NHL and EBV detection in peripheral blood, with a predominance of EBV type 1. Furthermore, children with NHL had higher viral load in their peripheral blood than EBV positive controls. The high level of EBV in blood may be relevant in predicting tumour burden, prognosis and possibly the outcome of chemotherapy. Moreover, the strong association between NHL and EBV may suggest that control subjects with high detectable levels of EBV in blood might be at higher risk of developing lymphoma.

Additional community-based studies should be done in order to identify the common EBV subtypes circulating in Tanzanian children and to predict their causal relationship with NHLs in Tanzania.

## Conclusions

BL is the most common childhood NHL subtype in north-western regions of Tanzania with rare CNS and bone marrow involvement. NHLs are not associated with HIV infection, but are strongly associated with EBV load in blood. Children with NHL at presentation had significantly higher frequency and higher levels of EBV in the peripheral blood than age matched controls. Overall, these findings suggest that EBV load in blood might be a diagnostic and prognostic marker for the onset and monitoring of NHL in African children. EBV detection in blood is less invasive and expensive than EBV detection in histological samples. Additional studies in larger populations are required to validate the diagnostic and prognostic value of EBV load in blood.

## Competing interests

The authors declare that they have no competing interest and this work was supported in part by Programma Integrato Oncologia (RO 4/2007).

## Authors’ contributions

RK; Designed the study, collected and analyzed data, interpreted the results and drafted the manuscript. NM; Designed the study, analyzed data, interpreted the results and reviewed the manuscript. PR; Designed the study, carried out pathological analysis of samples and reviewed the manuscript. EK; Designed the study, analyzed data, interpreted the results and reviewed the manuscript. BK; Analyzed data, interpreted the results and reviewed the manuscript. AD; Carried out molecular studies, interpreted the results and reviewed the manuscript. RP; Carried out molecular studies. DM; Designed the study, analyzed data, interpreted the results and reviewed the manuscript. All authors read and approved the final manuscript.

## Pre-publication history

The pre-publication history for this paper can be accessed here:

http://www.biomedcentral.com/1471-2431/13/4/prepub
